# Late Puerperal Infection

**Published:** 1887-06

**Authors:** 


Late Puerperal Infection.—As illustrative of the benefits of intra uterine irrigation and curetting for fever occurring late in the puerperal period, Dr. B. C. Hirst recently reported three cases to the Philadelphia Obstetrical Society. In these cases fever began on the fourteenth, eleventh and twelfth days, respectively, associated with fetid, muco-purulent discharges, patulous os, uterus large, pain and tenderness. Here the results of treatment justified the diagnosis that the source of infection was intra-uterine, as most cases of late infection are apt to be; also that some times the antiseptic intra-uterine irrigation is not sufficient to dislodge the offending cause, but that the blunt curette should be used, followed by irrigation.

